# Supporting Relatives Prior to Caregiver Burden—Preventive E-Mental Health Short Intervention for Family Members of Individuals with Parkinsonism in an Early Phase of the Disease: Protocol for a Feasibility Study

**DOI:** 10.3390/brainsci12040442

**Published:** 2022-03-26

**Authors:** Catharina Muente, Ann-Kristin Folkerts, Elke Kalbe, Franziska Thieken, Laura-Elisa Assmann, Maria Widritzki, Carsten Eggers, David Pedrosa, Marcel Wilhelm

**Affiliations:** 1 Department of Neurology, University Hospital Marburg, 35043 Marburg, Germany; thieken@staff.uni-marburg.de (F.T.); maria.widritzki@uni-marburg.de (M.W.); carsten.eggers@kk-bottrop.de (C.E.); pedrosac@staff.uni-marburg.de (D.P.); 2 Department of Medical Psychology Neuropsychology and Gender Studies and Center for Neuropsychological Diagnostics and Intervention (CeNDI), Faculty of Medicine, University Hospital Cologne, 50937 Cologne, Germany; ann-kristin.folkerts@uk-koeln.de (A.-K.F.); elke.kalbe@uk-koeln.de (E.K.); 3 Department of Clinical Psychology and Psychotherapy, Philipps University of Marburg, 35032 Marburg, Germany; assmannb@students.uni-marburg.de (L.-E.A.); marcel.wilhelm@uni-marburg.de (M.W.); 4 Center for Mind, Brain and Behavior (CMBB), Universities of Giessen and Marburg, 35032 Marburg, Germany

**Keywords:** Parkinson’s disease, early phase of Parkinsonism, non-caregiving relatives, burden, stress, quality of life, telemedicine, mental health, e-mental health, study protocol

## Abstract

Research on support for relatives of patients with Parkinsonism has mainly focused on caregivers, while preventive offers for non-caregiving relatives are lacking. Thus, the aim of this multicenter pilot study is to develop and assess the feasibility of a preventive psychosocial support program for relatives of patients with Parkinsonism. It specifically focuses on family members of patients who are in an early phase of the disease, are not currently caregiving, and have not yet developed distress symptoms. It includes a telemedicine-based, 6-week preventive psychological short intervention (PPSI). The main objective of this feasibility mixed-methods study is to specify the demand for an early, low-threshold, and low-cost short intervention and to collect feedback based on qualitative and quantitative data of *N* = 20 relatives. Secondary objectives are an evaluation of the effects of the intervention and an analysis of the study design. Future directions are to further develop the PPSI using these data. This study can serve as a basis for future randomized controlled studies on this intervention, which might fill an important gap in clinical supply.

## 1. Introduction

Parkinson’s disease (PD) is the second most common neurodegenerative disease, and its prevalence is expected to increase substantially in the coming years [1]. PD is mostly characterized by the cardinal motor symptoms of bradykinesia, rigidity, postural instability, and tremor [2]. In addition, several other motor and non-motor symptoms affect patients’ and their relatives’ lives. The non-motor symptoms comprise constipation, orthostatic hypotension, bladder dysfunction, sexual dysfunction, depression, anxiety, or cognitive impairment, among many others. Age is currently considered the main risk factor [3]. Neuropathologically, the disease is based on the degeneration of dopaminergic neurons of the substantia nigra pars compacta [3]. As the disease progresses, the motor and non-motor symptoms can increasingly restrict patients’ daily lives and may require a substantial amount of care [4].

The diagnosis of PD, but especially the rampant restrictions in daily life while the disease progresses, may entail major psychological implications for the affected person and relatives.

In many cases, loss of patients’ autonomy and difficulties coping with daily life (clothing, personal hygiene, nutrition, mobility, taking medication, etc.) may inevitably cause an increased level of dependence on the relatives’ support. A growing body of literature indicates that the symptom burden of PD patients and stress experienced by family caregivers strongly correlate [5,6,7,8]. 

Research indicates that many families try to ensure care in the familiar home environment for as long as possible. Especially at early stages, care is usually provided exclusively by close family members such as spouses, children, siblings, and also sometimes friends and neighbors [9,10]. With increasing disability, however, care provided by relatives alone may become insufficient [6], so that about 24% of PD patients receive professional care in their home environment and 19% permanently live in a care facility [11]. Related factors to the necessity of permanent care in a nursing home are increased age, pronounced motor symptoms, cognitive dysfunction, and neuropsychiatric impairments, but especially psychotic symptoms [6,10,12]. In addition, safety concerns and caregiver-related factors such as managing changes in caregiver health play a crucial role in the institutionalization of PD patients [4]. 

Family members may experience this role change within disease progression from spouse/relative to caregiver to mere spectator due to changes in their social position, which often encompasses conflicts between patient and relatives [13]. Due to the multidimensional character of the burden, stressful situations of relatives are often not recognized in time, and relatives are therefore also considered as “invisible patients” [14]. The burden of relatives often leads to serious effects on emotional, social, physical, and financial aspects of life as well as the ever-increasing scarcity of time [14,15]. It should also be noted that the burden might not only begin with the diagnosis but with the first, initially unspecific symptoms—even for the relatives. In addition, fear of Parkinsonism/PD progression leads to a further experience of stress [16], so that overall, a significantly increased risk of a reduced quality of life and the development of depression may be the consequences [14,17,18]. However, the sample of our study does not consist exclusively of PD patients but also includes patients with Parkinsonism and their relatives to design a comprehensive support program for relatives with similar experiences.

It is already known that caregivers of patients with Parkinsonism often suffer from severe psychological stress in the late phase of the disease. The burden on caregivers increases with disability and symptoms of Parkinsonism. This is especially relevant for mental health problems such as depression, hallucinations or confusion, and falls [19]. The duration and severity of the disease correlates with the burden on the relatives [20]. They have an increased risk of psychiatric morbidity and persistent distress [21]. However, little is known about the mental attitudes and burdens of family members in the early phase of the disease [14,18]. 

The main study population does not yet correspond to the conventional definition of family caregivers, because they are not yet giving informal care. Therefore, the term “non-caregiving relatives” will be used. The sample thus includes partners of patients with Parkinsonism in the early phase of the disease (Hoehn and Yahr I–II) who live together in one household and who are not yet involved in caregiving.

Despite the growing research on the burden on caregivers of patients with Parkinsonism, there is still limited evidence on interventions for non-caregiving relatives [22]. Focusing on non-caregiving relatives is, however, of immense importance to support them as early as possible and to alleviate burdens and avoid severe strain [23]. To the best of the authors’ knowledge, there are no prevention support programs for the target population of the study. However, there is evidence of programs for diseases for which relatives are at increased risk, such as strain, (stress,) depression, and anxiety [24,25]. 

Existing support programs for family members of people with PD are often educational and provide therapeutic support but are mainly aimed at family members of patients in the late phase of the disease who are already involved in the care of the person with PD. These programs are often aimed at both PD patients and their caregivers taking part together [18,26,27,28]. This study aims to evaluate an intervention that is only aimed at the relatives.

The support program will be conducted online. Since the COVID-19 pandemic has led to digitalization in telemedicine in many areas as well as the field of neurology and the care of PD patients [29]. Nevertheless, the availability of online support programs is not yet widespread. However, approaches have been developed to examine telephone-based self-support groups [30], online teaching programs for patients with Parkinsonism in the late stage [31], and educational programs offered by healthcare professionals for caregivers [32]. Because an E-health approach is still quite uncommon, there is little data on the effectiveness of support services for caregivers. However, effectiveness has already been demonstrated in the context of preventive support for relatives in other related fields such as mental health and dementia [33,34,35].

Nevertheless, there is a lack of preventive telemedical offers for non-caregiving relatives of Parkinson’s patients [36]. The term ‘e-mental health’ refers to technological approaches that maintain or promote psychological health [37]. 

Therefore, a preventive psychological short intervention (PPSI) for non-caring relatives is to be developed within the scope of the study.

This study explicitly addresses relatives who are not (yet) involved in family care. 

The intervention itself is solely aimed at the non-caregiving relatives and intends to strengthen resources, thereby fostering empowerment in the course of a progressive disease. Topics of the intervention, which follows a cognitive–behavioral therapy approach, are, on the one hand, the visualization and activation of relatives’ social network of relationships, and, on the other hand, specific exercises with relatives of people with Parkinsonism to empower dealing with stressful situations possibly arising in the future. The intervention is offered online and allows participation from home to facilitate implementation into relatives’ everyday life. The key question is, therefore, a threefold feasibility analysis:

1. Feasibility of the intervention: the feasibility will be assessed via acceptability, fit and subjective benefit.
2. Feasibility of the manual (adherence): it is to be assessed whether all planned aspects of the intervention have been discussed or if there are other topics that will be brought up by the relatives.
3. Feasibility of the study design: investigating the feasibility of the study design means critically reflecting on the mixed-methods approach, the process of a baseline measurement with post and follow-up assessments, as well as the validity of the questionnaire selection and the structure of the interview guide. The data from questionnaires will be used for exploratory analyses.

Against this background, the primary aim of this multicenter pilot trial following a mixed-methods approach is to develop and assess the feasibility of a preventive psychological short intervention to relatives of patients with Parkinsonism at early stages. In addition, the secondary aims are to investigate the effects and manual adherence of the intervention.

## 2. Methods and Study Design

### 2.1. Methods

This study will be conducted at two different locations in Germany, the Department of Neurology of the University Hospital in Marburg (study number: 175/20) and the Department of Medical Psychology/Neuropsychology and Gender Studies of the Faculty of Medicine and the University Hospital Cologne of the University of Cologne (study number: 21-1061). Ten patients and ten relatives will be included as dyads per site so that a total of 20 relatives and 20 patients with Parkinsonism will be included. Recruitment will take place separately at both sites. At the Marburg site, patients and their relatives will be recruited via an internal recruitment system of the Department of Neurology at the University Hospital that lists patients with Parkinsonism who are generally interested in participating in studies. In Cologne, recruitment will be ensured via lists of study participants from previous studies. Further, e-mail distribution lists of self-help groups will be used to increase the recruitment numbers.

Inclusion criteria for patients are:

(1) Patients with a clinical diagnosis of Parkinsonism according to the Movement Disorder Society (MDS) criteria [2];
(2) Patients aged ≥ 18 years;
(3) Hoehn and Yahr stages I–II (OFF-state).

The exclusion criteria for the patients are:

(1) Hoehn and Yahr stage ≥ III (OFF-state);
(2) No care requirements and/or outpatient or inpatient care;
(3) Suspected dementia according to physicians’ reports and (third-party) anamnesis.

Inclusion criteria for non-caregiving relatives are:

(1) Partners of people diagnosed with Parkinsonism, living together in the same household;
(2) Age ≥ 18 years;
(3) Not involved in informal caregiving of the subject with Parkinsonism;
(4) Willingness to use telemedicine, availability of a PC or tablet with stable internet connection;
(5) No cognitive impairment (operationalized by the Montreal Cognitive Assessment [MoCA] [38], Blind Version ≥ 19; the MoCA Blind, which is suitable for online administration and therefore used here, is reduced to 22 items and converted back to 30 points maximum. A total of 19 points in the MoCA Blind version correspond to 26 points in the original MoCA “Full”);

Exclusion criteria for the relatives are:

(1) Own caregiving requirement;
(2) Severe depression (operationalized by the Beck’s Depression Inventory-II [BDI-II] ≥ 29 points) [39].

### 2.2. Study Design

**Figure 1:**
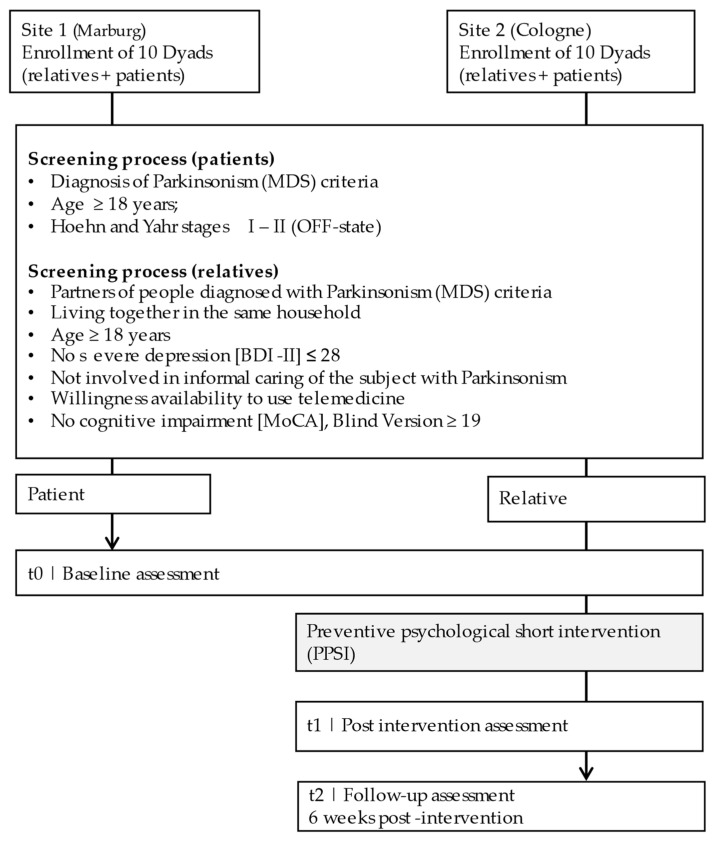
Schematic course of the study for each dyad.

The patients and their relatives will be informed verbally and in writing about their participation in the study. By signing the consent form, they are included in the study. Figure 1 depicts the three time points of measurement: baseline (t0), post-testing immediately after the end of the intervention (t1), and follow-up (FU) at 6 weeks post-intervention (t2). The intervention is to be understood as a preventive psychological short intervention for relatives. Patients will not participate in the intervention. No remuneration will be paid to the participants.

### 2.3. Intervention Protocol

The PPSI includes three sessions of maximum 50 min at intervals of max. 2 weeks and is to be conducted in an online meeting with the relative and a trained and supervised advisor (at least B.Sc. in Psychology or related discipline). A licensed therapist (MW) trains new advisors on how to use the manual and supervises the PPSI on a weekly basis. The Red Medical platform (https://www.redmedical.de/ accessed on 3 March 2022) ensures the General Data Protection Regulation (GDPR)-compliant implementation of video consultations with overall end-to-end encryption so that conversations remain strictly confidential and cannot be accessed by third parties. The cognitive–behavioral therapy-based intervention consists of three sessions, which include the following elements: standardized elements with knowledge transfer, personalized elements addressing the individual’s perspective, and exercises to apply the knowledge on an individual basis. As support, working sheets will be used, which also serve for future implementation of the intervention elements in the study participants’ everyday life.

At a content level, the mental health of the relatives is the focus of the intervention. Each session deals with a psychological focus area. The areas are based on protective factors in prevention of mental disorders, e.g., ability to manage stress, empowerment, and social participation [40]. The most relevant areas were selected via expert panels (CE, AF, EK, CM, FT, and MW) with regard to the most relevant issues in giving care to patients with Parkinsonism. The overarching goal is to provide self-management strategies for relatives of PD patients in the sense of “empowerment”, which can be applied at a later stage of the disease.

#### 2.3.1. Session 1—Understanding the Situation of the Relatives, Stress-Management Strategies

The first session focuses on establishing a relationship between the trained advisor and the relative as well as on exploring the personal situation and life circumstances of the relative. This includes asking about potential individual fears of disease progression. In addition, goals, wishes, and expectations towards the short intervention are discussed with the relatives. Then, a psychoeducational unit on stress management follows, containing the transfer of knowledge on the transactional stress model according to Lazarus [41]. Possible individual stressors are identified, and coping strategies are worked out in a problem-oriented or emotion-oriented way. Key questions: “what changes to the situation/action can be taken to reduce (any future) stress?” (problem-oriented coping). “How can the relative deal with the situation better?” (emotion-oriented coping).

#### 2.3.2. Session 2—Social Network and Self-Management Strategies

The second session is designed to promote self-management strategies and individual resources as well as autonomy [14,42]. First, the so-called airplane metaphor is discussed with the relatives to individually design the topic of self-care [43]. The core idea of the airplane metaphor is that every passenger in the airplane is asked as part of the safety instructions to first put on the oxygen mask themselves in case of a loss of air pressure and then to help other people [43].

Afterward, concrete methods to increase self-care will be discussed: the social (and, if necessary, professional) environment of the relatives is explored and noted in a specially provided page of the workbook. Then, with self-care in mind, it is discussed to what extent this “support network” could become active in a supportive way in the further course of the disease [9]. With the help of a visualized list, it can be possible at an early stage to identify and (re-)activate important people to the “inner circle” of the relatives.

This is followed by a unit on values orientation. Here, based on a list of values previously filled out by the relatives, they work out which personal values they feel to be important [44]. The goal is illustrated by the so-called bus driver metaphor [45]. In this imagination exercise, the relatives are asked to imagine themselves as bus drivers and to transport both positively and negatively associated passengers. The passengers symbolize negative or positive experiences and worries in dealing with Parkinsonism. Especially in the case of increasing stress (e.g., due to progression of the disease), it is helpful to activate the relatives’ personally important values and encourage them to adapt their behavior accordingly [45]. To act in accordance with personal values helps to stay psychologically flexible while experiencing interfering negative thoughts, emotions, and bodily sensations [46]. For example, a relative ruminating about Parkinsonian symptoms of their spouse will sometimes feel helpless. It could then be comforting to do something meaningful to them personally, e.g., helping others in similar situations (value: helpfulness), making the best out of the situation with the partner (relationship), or focusing on their own career (success). The relatives should be encouraged to live by their own values, regardless of their partners’ illness.

#### 2.3.3. Session 3—Conception of Further Strategies Based on Personal Values

In the third and last session, the aim is to summarize the two previous sessions and record the contents discussed for later use. In this session, a concise strategy for dealing with worries will be conveyed through an exercise on the non-judgmental perception of thoughts [47]. This can be a useful skill to describe negative feelings more neutral and hence break the vicious circle of rumination. In addition, as a conclusion to the work on values, the participants should practice aligning their own action planning with a personal value. The session concludes with discussing the personal take-home messages, with participants summarizing what they have gained from the conversations.

### 2.4. Outcome Measures

This pilot study follows an explorative mixed methods approach focusing on feasibility aspects and effects of the intervention using both quantitative and qualitative data. Data will be collected from both relatives and patients to capture the factors influencing the burden of relatives in the early phase of the disease. It is known from previous studies on the late phase that, in addition to the patient’s health status and the severity and duration of the disease, other patient-related aspects influence caregiver burden [48]. For the relatives, mental health, sociodemographic factors, and personality aspects, among others, are considered predictors of burden [36]. The data collection is thus intended to provide a diverse and complete picture of the burden on relatives in the early stages of Parkinsonism.

#### 2.4.1. Effects of the Intervention

To determine whether the intervention has led to health- and stress-management-related changes in the relatives, standardized questionnaires and psychometric tests will be conducted at three time points of measurement (t0, t1, t2). The questionnaires address various parameters: personality, sense of burden, depression, cognition, quality of life, coping strategies for stress, self-management, expectation of therapy, and subjective benefit (see Table 1 for details).

Montreal Cognitive Assessment (MoCA) Blind 8.1; [38]; Parkinson’s disease caregiver burden questionnaire (PDCB) [49]; Zarit Burden Interview (ZBI) [50]; Beck’s Depression Inventory (BDI-II) [39,51]; Coping Inventory for Stressful Situations (CISS) [52]; Measurement of health-related quality of life (EQ-5D) [53]; Questionnaire for the assessment of resources and self-management skills (FERUS) [54], Therapy Expectancy and Evaluation Questionnaire (PATHEV) [55]; Credibility/Expectancy Questionnaire (CEQ) [56]; The Parkinson’s Disease Questionnaire (PDQ-39) [57]; Big-Five-Inventory 10 (BFI 10) [58].

After completion of data collection, quantitative data will be analyzed using statistical software IBM SPSS Statistics 27. Detailed descriptive analyses as well as paired-samples *t*-tests and Mann–Whitney-U-test, respectively, will be conducted, according to the distribution of the data. Subjects who drop out of the study will be included in the analysis in the sense of an intention-to-treat analysis. Table 1 provides an overview of the questionnaires and the neuropsychological test battery.

#### 2.4.2. Feasibility Analysis of the Intervention

The qualitative data will provide a more detailed insight into the perspective of relatives of patients with Parkinsonism as potential future primary caregivers. Qualitative, semi-structured interviews will be conducted at two time points via an online consultation with relatives immediately after the intervention (t1) and 6 weeks post-intervention (t2). This part of the feasibility analysis is structured by the three outcome aspects of acceptance, fit, and subjective benefit with corresponding operationalizations and queried in the interviews. The interview guide contains questions on acceptance, fit, and personal benefit of the intervention.

All interviews at t1 and t2 will be recorded and transcribed following data collection and stored for 10 years. During transcription, the ruleset suggested by Dresing and Pehl [59] will be followed. The spoken content is prioritized accordingly, while information on para- and non-verbal events are omitted. The MAXQDA 2020.4 software will be used for qualitative content analysis according to Kuckartz [60] using a combined model of deductive (a priori) and inductive coding (on the text material).

#### 2.4.3. Feasibility of the Intervention Manual

Not only the feasibility and impact of the intervention but also the manual adherence will be assessed quantitatively using an evaluation scheme developed for this purpose. Each section will be listed to independently rate whether the respective topic was addressed, as well as whether the worksheets provided were discussed. The scale consists of 14 items (10 topics, 4 worksheets) that are answered on a 4-point Likert scale ranging from 0 (not addressed) to 3 (fully addressed). A sum score will be obtained at the end to quantify adherence. Further topics addressed outside the manual will be noted. The manual adherence ratings will be completed by an independent rater using tape recordings of the sessions. This also allows noting deviations from the intervention time (50 min per session). The participant’s audio track will not be recorded and evaluated; only the advisor’s track will be recorded via the microphone output. However, all participants give their prior consent to these recordings.

With the help of feedback from the relatives, the feasibility of the intervention is evaluated. Therefore, the workbook also functions as an intervention diary. Before each session, the mood and motivation of the relatives are recorded. Except for the first session, they are asked what they remember of the previous session and if it was of any use to them. After each session, the relatives’ mood and satisfaction with the session are assessed by asking them how helpful they found the content and delivery of the intervention. The data are collected using a six-point Likert scale ranging from 0 (not at all) to 5 (very much).

#### 2.4.4. Feasibility of the Study Design

To examine the feasibility of the study, the test battery will also be critically examined regarding length and expected benefit. The feasibility of the study design will be discussed in an expert panel, considering the results of the interviews and the completed questionnaires. In addition, a dropout analysis will be conducted to evaluate the reasons for participants dropping out of the study.

An overview of the feasibility analysis is summarized in Table 2.

## 3. Discussion

Currently, there is limited evidence on the feasibility and effects of interventions for family members of people with Parkinsonism. The existing offers for relatives mostly have a psychoeducational orientation and refer to relatives of patients in a more advanced phase [18,26,27]. There are also approaches offering CBT treatments [28], but these can be quite time-consuming and do not have a preventive focus. Digital support programs based on telemedicine are currently still very rare, but they exist, for example, as a model of a telephone self-help group [30]. However, there are no outreach or preventive programs that specifically address relatives of patients in the early phase of the disease. The aim of the present study is, therefore, to develop a PPSI for relatives of people with Parkinsonism and to determine whether the target group accepts it, and the feasibility regarding its preventive approach and digital implementation is of particular interest. Furthermore, the fit of PPSI and the relatives’ needs, as well as their subjective benefit from the intervention in the current state, should give valuable feedback on which topics to add or remove in a future modified version of the preventive support offer.

Due to the multidimensional character of the burden that relatives of people with Parkinsonism are confronted with, and the rather high sense of stress at the time of diagnosis, implementing a PPSI with similar objectives appears to be an important approach.

Notwithstanding more methodological concerns about the feasibility of such an intervention, there are also technical questions risen by the study presented. It is essential to consider that not all people have access to the technical prerequisites or have the ability or the will to use them. Although technical devices increasingly bridge the existing spatial distance [61], in some areas, access to in-person healthcare services and digital resources are more limited than in others. It remains to be elucidated if the expansion of digital resources, e.g., in rural areas, may counteract these limitations and if our intervention, while disease stages are within a mild to moderate range, can somehow contribute to supporting relatives. Despite the lack of closeness due to familiarity and informal communication within our study, we aim at providing a short and digital intervention that will be easily implemented into the daily life of the relatives [62]. Especially during the COVID-19 pandemic, the easy access from home is a great advantage of the PPSI. In addition, digital communication enables people from rural areas to be reached more easily.

As this pilot study is to be understood as a starting point for further preventative approaches, there are several limitations. Due to the small number of participants (*n* = 20), it is not possible to make reliable statements about the impact of the intervention. Predicting whether a preventive approach can sustainably support relatives to avoid stress and burdens in the long term and in advance would only be possible with a larger study population and a longitudinal design covering several years. Another limitation is that the study period of the intervention is probably too short to capture the effects on the daily life of the family members in the further course of the disease. Therefore, the study will serve as preparation for a large-scale, prospective, randomized, longitudinal controlled clinical trial.

## 4. Conclusions

This study aims to test a preventive e-mental health offer for relatives of people with Parkinsonism. It will be apparent whether the target group accepts this offer and what suggestions and feedback are revealed in the evaluation. With the help of feedback, especially from the interviews, the psychosocial intervention will be further developed to ensure that the offer is oriented according to the needs of the relatives. After evaluating the pilot study, a modification of the intervention and the study design is planned, relying on the findings. Based on this, a multicenter randomized, controlled trial will be implemented.

## Figures and Tables

**Table 1:** Core outcome set and measurement instruments.

Measurements	Outcome	Baseline	Post Intervention	Follow-Up (6 Weeks)
**Patients**				
Sociodemografic data	Personal data	x		
Big-Five-Inventory 10 (BFI)	Personality	x		
PDQ-39	Health status regarding PD	x		
Beck’s Depression inventory (BDI-II)	Depression	x		
**Relatives**				
Sociodemografic data	Personal data	x		
Montreal Assessment (Moca) Blind 8.1	Cognition	x		
Parkinson’s disease caregiver burden questionnaire (PDCB)	Burden on family carergiver	x	x	x
Zarit Burden Interview (ZBI)	Burden on family carergiver	x		
Beck Depression inventory (BDI-II)	Depression	x	x	x
Health-related quality of life (EQ-5D)	Health-related quality of life	x	x	x
Coping Inventory for Dealing with Stressful Situations (CISS)	Coping strategies for stressful situations	x	x	x
Questionnaire on Health-Related Resources and Self-Management Skills (FERUS)	Health-related self-management strategies	x	x	x
Therapy Expectancy and Evaluation Questionnaire (PATHEV)	Therapy Expectancy	x	x	
Big-Five-Inventory 10 (BFI)	Personality	x		
Subjective Benefit (Credibility/Expectancy Questionnaire (CEQ))	Subjective Benefit		x	
Semi-structured Interview	Feasibility of the intervention		x	x

**Table 2:** Feasibility analysis.

Outcome	Measurement Instruments	Operationalisation
Feasibility of the Intervention		
Acceptance	Interview	Willingness of the target group to participate
		Reasons for participation
		Evaluation of the digital intervention
Fit	Interview	Scope of the intervention
		Effort for the relatives
		Choice of timing
		Suggestions for optimization
		Overall assessment
Subjective Benefit	Interview	Particularly helpful aspects
		Psychosocial changes that the relatives notice about themselves
Evaluation of the intervention	Questionnaire (Filled in by the relative)	Questionnaires for the evaluation of each session
Feasibility of the intervention manual		
Adherence	Questionnaire (Filled in by Advisor)	Assess whether all intended aspects of the intervention have been discussed. A maximum of 14 points can be achieved
Feasibility of the study design		
Quality criteria	Expert panel	Quantiative: Validity, reliability, objectivity Qualitative: Transparency, intersubjectivity, range Drop-Out-Analysis
